# Formulation and In Vitro Evaluation of Oral Capsules and Suspension from the Ethanolic Extract of *Cola nitida* Seeds for the Treatment of Diarrhea

**DOI:** 10.1155/2021/6630449

**Published:** 2021-06-29

**Authors:** Fredrick W. A. Owusu, Christiana O. Asare, Philomena Enstie, Ofosua Adi-Dako, Genevieve Naana Yeboah, Doris Kumadoh, Amanda Tetteh-Annor, Edem M. Amenuke, Mordey Karen

**Affiliations:** ^1^Department of Pharmaceutics, Faculty of Pharmacy and Pharmaceutical Sciences, Kwame Nkrumah University of Science and Technology, Kumasi, Ghana; ^2^Department of Pharmaceutics, School of Pharmacy, Central University, Miotso, Ghana; ^3^Department of Herbal Medicine, Faculty of Pharmacy and Pharmaceutical Sciences, Kwame Nkrumah University of Science and Technology, Kumasi, Ghana; ^4^Department of Pharmaceutics and Microbiology, School of Pharmacy, University of Ghana, Ghana; ^5^Centre for Plant Medicine Research, Akuapem-Mampong, Ghana

## Abstract

Management of diarrhea has evolved over the years from relatively inadequate interventions in the early years to more successful physiological approaches. The use of herbal medicinal products and supplements has grown significantly over the past three decades, with more than half of the global population depending on it for some aspect of their primary health care needs. This study is aimed at formulating solid and liquid oral dosage forms of the ethanolic extract of *Cola nitida* seeds for the treatment of diarrhea. The flow property of the dried ethanolic extract was determined and subsequently formulated into granules for encapsulation. The ethanolic extract was also used in formulating an oral suspension. Pharmacopeia tests such as uniformity of weight, disintegration, drug content, and dissolution were carried out on the formulated capsules. The formulated suspension was also assessed using the following parameters; viscosity, flow rate, drug content, dissolution, sedimentation rate, and sedimentation volume. The dried ethanolic extract and formulated granules exhibited good flow properties. The formulated capsules exhibited optimal *in vitro* release of extract (>90% after 45 minutes) and passed the uniformity of weight, disintegration, and drug content tests. The formulated suspension also passed the drug content test and had a good sedimentation rate, sedimentation volume, and flow rate. The formulated suspension also exhibited pseudoplastic flow, optimal viscosity, and a good *in vitro* release profile (>90% after 45 minutes). Capsules and suspension of the ethanolic extract of *Cola nitida* seeds have been successfully formulated and can be used as standard dosage forms for the management of diarrhea.

## 1. Introduction

Diarrhea can be defined as the passage of three or more loose or watery stools over a 24-hour period or more frequent passage of stools than normal for an individual. A loose stool is one that would take the shape of a container [1]. Diarrhea also occurs when secretion of water into the intestinal lumen exceeds absorption. This disease is one of the most common clinical signs of gastrointestinal disease but can also reflect primary disorders outside of the digestive system. Diarrhea affects both adults and children with debilitating effects on both categories of patients. However, children are more susceptible to the effects of the disease as compared to adults. This disease is also prevalent in developing countries [2–4].

About 90% of all diarrheal death occurs in south Asia and sub-Saharan Africa due to poor environmental conditions and weaker health care systems [1, 4, 5]. In Ghana, it accounts for 25% of mortality in children under five with more than 9 million episodes occurring annually. Pharmacological and nonpharmacological interventions can be used in the management of diarrhea. Due to the relatively swift progression of the disease, usually, patients are offered pharmacological treatments and supported with nonpharmacological interventions. Many orthodox medications have been developed to treat diarrhea. However, due to cost and lack of accessibility to orthodox medications, patients in developing countries such as Ghana, are still dying from this disease. For this reason, the World Health Organization encourages studies into indigenous herbal medicinal plants that can be used to manage and treat diarrhea and subsequent formulation of these medicinal plant extract into standardized dosage forms which will be safe, effective, and convenient for patients. In Ghana, several medicinal plants such as *Justicia flava* (Hiern), *Lannea welwitschia*, *Anacardium occidentale* L., *Mangifera indica* L., and *Cola nitida* have been documented as being used folklorically in the treatment of diarrhea with *Cola nitida* being common amongst most of the tribes in Ghana [6, 7].

*Cola nitida*, a member of the Sterculiaceae family, is native to rainforests in tropical West Africa and is generally referred to as kola nut or bitter kola. Traditionally, the leaves, twigs, flowers, fruit follicles, seeds, and the bark of *Cola nitida* are used to prepare a tonic as a remedy for dysentery, coughs, diarrhea, vomiting, and chest pains [8]. Scientific studies carried out by Doe et al. [9] determined the pharmacological dose and confirmed the antidiarrheal effect of the ethanolic extract of *Cola nitida* seeds. However, to the best of our knowledge, there are no standardized dosage forms of this extract, and as such, prescribers and patients are not able to utilize it in the management and treatment of diarrhea. Thus, this study seeks to formulate solid (capsules) and liquid (suspension) oral dosage forms of the ethanolic extract of *Cola nitida* seeds for the treatment and management of diarrhea. These dosage forms will provide safe and convenient alternatives for patients and prescribers in using the ethanolic extract of *Cola nitida* seeds to treat diarrhea.

## 2. Materials and Methods

### 2.1. Materials

The materials used are *Cola nitida* seeds (Nima, Ghana); maize starch (Anhui Sunhere Pharmaceuticals, China); lactose, polyethylene glycol (PEG), and tragacanth (Sigma Aldrich); and 90% ethanol, chloroform, benzoic acid, distilled water, and 0.1 MHCL (Department of Pharmaceutics, Central University). All other chemicals and reagents used in this study were of analytical grade.

### 2.2. Methods

#### 2.2.1. Sample Collection and Extraction

*Cola nitida* seeds were purchased from a local market in Nima, Ghana, and authenticated by the Department of Pharmacognosy, School of Pharmacy, Central University, Ghana. The seeds were carefully inspected to make sure that each seed was suitable for the extraction process. Ethanolic extraction of the *Cola nitida* seeds was carried out as described by Doe et al., 2019 [9].

#### 2.2.2. Evaluation of Flow Properties of the Dried Extract

Indirect method of characterizing flow properties of powders as described by Aulton and Taylor [10] was used.

### 2.3. Formulation of *Cola nitida* Suspension

An amount of 0.1 g of benzoic acid was accurately weighed using an analytical balance into a clean mortar. Two (2) g of tragacanth powder was weighed into the mortar and triturated using a pestle. An amount of 6.5 g of the *Cola nitida* extract was then added to the mixture with continuous trituration followed by 50 mL of chloroform water. The resulting product was transferred into the suspension bottle and distilled water added to the 100 mL mark (each 5 mL contained 0.33 g of *Cola nitida* extract). The suspension was covered and labelled appropriately.

### 2.4. Preparation of *Cola nitida* Granules

Starch (1.0 g), lactose (2.74 g), and *Cola nitida* extract (9.75 g) were weighed into a clean mortar using the method of doubling the bulk [10]. Tragacanth mucilage (10% *w*/*v*) was prepared in another mortar by measuring 10 g of tragacanth and levigating with 55 mL of water. It was then transferred quantitatively into an amber bottle. Water was added to make up to the 100 mL mark. The tragacanth mucilage was then added to the powdered mixture until a damp mass was formed. The damp mass was screened through mesh no. 8 and then dried in a hot air oven at 40°C. The dried granules were then sieved with mesh no. 16 and stored in airtight containers pending further analysis.

### 2.5. Evaluation of *Cola nitida* Granules

Indirect methods were used to characterize the flow properties of the formulated granules, namely, bulk density measurements (Hausners ratio and Carr's index) and angle of repose (fixed height method) [10].

### 2.6. Formulation of *Cola nitida* Capsules

Capsule size 0 was used in encapsulating the granules. Fifty capsules were filled simultaneously using the capsule filling machine (each capsule contained 0.170 g of *Cola nitida* extract). The capsules were then packaged and appropriately labelled pending further tests.

### 2.7. Quality Assessment of Formulated Capsules

#### 2.7.1. Uniformity of Weight Test

Twenty capsules were randomly selected, and each was weighed including an empty capsule shell. The total weight of the content of each capsule, average weight of content per capsule, and the percentage deviation of individual content weights from the mean were calculated [11, 12].

#### 2.7.2. Disintegration Test

Disintegration time for the formulated capsules was assessed using the T-TD-2 disintegration apparatus. Six capsules were placed in six tubes of the basket rack assembly, and the apparatus was operated using distilled water at 37 ± 2°C. The capsules were observed, and the time taken for complete disintegration of all capsules was recorded [11, 12].

#### 2.7.3. Uniformity of Drug Content Test

*(1) Preparation of Calibration Curve*. Serially diluted solutions of the ethanolic extract in 0.1 MHCL was used to determine the maximum wavelength of absorption (320 nm). Using serial dilution, a stock solution was then diluted to produce concentrations of 0.03% *w*/*v*, 0.0025% *w*/*v*, 0.02% *w*/*v*, 0.015% *w*/*v*, and 0.010% *w*/*v*. The UV spectrometer was set to 320 nm and then used to run samples from each of the solutions using 0.1 M HCL as the blank sample. The absorbances recorded were plotted against their corresponding concentrations to obtain a standard calibration graph and the equation of the line determined [13–15].

*(2) Drug Content*. Ten capsules were selected randomly, each was emptied and 50 mL of 0.1 M HCL added. The mixture was filtered, and 0.1 M HCL was used to top it up to 100 mL. The Drawell's UV spectrometer was used in measuring the absorbances at 320 nm after serially diluting the solutions. The recorded absorbance was then inserted into the calibration equation and the percentage content determined for each capsule [11, 13–15].

#### 2.7.4. *In Vitro* Dissolution Study of Capsules

In vitro dissolution study was done using USP type II paddle dissolution apparatus. In 900 mL of 0.1 M HCl, the release medium was transferred into each of the six dissolution vessels. The water jacket was heated to 37 ± 2°C to represent the body's temperature and a randomly selected capsule (fitted with a sinker), dropped into the dissolution medium. An aliquot of the sample was withdrawn at regular time intervals (5, 15, 30, 35, 40, 45, and 60minutes), and the same volume of 0.1 M HCL was used to replace the volume withdrawn. The replacement process was done to maintain sink conditions. The samples were filtered and analyzed with the UV spectrometer at 320 nm. The absorbances obtained were inserted into the calibration equation to obtain the amount of drug released at each time point. A graph of cumulative drug released against time was then plotted to obtain the dissolution profile of the formulated capsules in 0.1 M HCL [11, 13–15].

### 2.8. Quality Assessment of Formulated Suspension

#### 2.8.1. Appearance Test

The formulated suspension was observed carefully at weekly intervals for 4 weeks for physical changes such as aggregation, caking, and crystal growth formation [16, 17].

#### 2.8.2. Sedimentation Volume and Rate

The sedimentation volume of the suspension was determined by measuring the volume of the sediments in 50 mL of the formulated suspension, on weekly basis for 4 weeks. The sedimentation volume (*F*) was calculated using the formula *F* = Vu/Vo (1) where Vu is the ultimate volume of sediment and Vo is the original volume of sediment before settling occurred. Triplicate determinations were done, and the mean and standard deviations were calculated. From the *F* values obtained, a graph of sedimentation volumes (Vu/Vo) against time was plotted, from which the sedimentation rate was calculated [16–18].

#### 2.8.3. pH, Viscosity, and Rheology

The pH of the formulated suspension was determined when it was freshly prepared (0 day) and then weekly for 4 weeks. The determinations were done in triplicates, and their means and standard deviations recorded. The viscosity of the formulated suspension was measured using the HBDV-I viscometer at 27°C with spindle number 2 and shear speeds of 10, 30, 60, and 90 rpm [16–18].

#### 2.8.4. Flow Time and Apparent Viscosity

Using a stopwatch, the time required for the formulated suspension to flow through a 10 mL pipette was determined. The apparent viscosity (*η*) was determined using the following equation: *η* (mL/s) = volume of pipette (mL)/flow time(s). This procedure was carried out weekly for 4 weeks and in triplicates [16–18].

#### 2.8.5. Redispersibility

The formulated suspension (50 mL) was transferred into capped cone tubes and evaluated for redispersibility at weekly intervals for 4 weeks, by turning it through a 180-degree cycle. Redispersibility was recorded as the number of inversions (strokes) required to completely resuspend the formulation in the cone tube [16–18].

#### 2.8.6. Uniformity of Drug Content Test

Five (5) mL of the formulated suspension was diluted with 45 mL of 0.1 M HCL, filtered, and topped up to 100 mL using the same solvent (0.1 M HCL). The Drawell's UV spectrometer was used in measuring the absorbances at 320 nm. The recorded absorbance was then inserted into the calibration equation and the percentage content determined. This was repeated nine times [11, 16–18].

#### 2.8.7. *In Vitro* Dissolution Study of Suspension

The USP type II paddle dissolution apparatus was used, and 900 mL of 0.1 M HCL heated to 37° ± 2°C by the water jacket. The dissolution paddle was set to a speed of 50 rpm, and 5 mL of the prepared suspension was transferred into the dissolution medium. After 5, 15, 30, 35, 40, 45, and 60 minutes, an amount of 20 mL of the dissolution medium was withdrawn and filtered, and the absorbance at 320 nm was determined using Drawell's UV spectrometer. The volume (20 mL) withdrawn was replaced with same volume of 0.1 M HCL to maintain sink conditions. The absorbances obtained were inserted into the previously determined calibration equation to obtain the amount of drug released at each time point. A graph of cumulative drug released against time was then plotted to obtain the dissolution profile of the formulated capsules in 0.1 M HCL [11, 16–18].

## 3. Results and Discussion

### 3.1. Flow Properties of Dried Extract and Granules

Flowability is most commonly assessed through measurements of angle of repose, compressibility index, Hausner's ratio, flow rate through an orifice, and others. The powdered extract and formulated granules exhibited good flow properties with Hausner's ratios of 1.16 ± 0.01 and 1.15 ± 0.03, respectively, Carr's index of 15 ± 0.04% and 14 ± 0.02%, respectively, and angle of repose of 27.25 ± 1.40° and 26.25 ± 1.20°, respectively. Good flow properties of granules will increase the uniform filling of capsules and enhance the ease of encapsulation [11, 13].

### 3.2. Evaluation of Formulated Capsules

#### 3.2.1. Weight Uniformity of Formulated Capsules

The average weight of the formulated capsules indicates that it is under the category of less than 300 mg which means for the capsules to pass this test, a maximum of 18 capsules should not exceed ±10.0%. The percentage deviation should also not exceed a limit of ±20.0% for a maximum of 2 capsules [12]. According to Table 1, not more than 18 capsules exceeded the percentage deviation limit of ±10.0%, and not more than 2 capsules exceeded the limit of ±20.0% which indicates that the capsules passed the uniformity of weight test. This means that each formulated capsule contains the stipulated amount of drug substance and excipients with little variation and this confirms that the encapsulation process was well carried out.

#### 3.2.2. Disintegration Time of Formulated Capsules

According to [11], the acceptable disintegration time for hard gelatinous capsules should not be more than 30 minutes.  Table 2 shows the average time for the disintegration test as 2.085 ± 0.05 mins. The results indicated that the capsules disintegrated properly within standard time range, and thus, the drug particles will be amply released for subsequent dissolution [13].

#### 3.2.3. Drug Content

A linear calibration plot with *R*^2^ of 0.9991 was obtained which shows a good relationship between the concentration and absorbance. This shows that absorbance values of *Cola nitida* solutions at *λ*max 320 nm can be used to evaluate the active principles present in the formulation [19]. The calibration curve produced an equation of *y* = 27.48*x* + 0.0002 which was used subsequently to calculate the percentage drug content for ten randomly selected capsules. In accordance with [11], the amount of drug substance calculated should be within the range of 85.0% to 115.0% in nine of the dosage units assayed with no unit out of the range of 75.0% to 125.0%. All ten of the capsules had their percentage drug contents within the standard range (Table 3). This indicates that the formulated capsules contained the required amount of the plant extract. This further corroborates the fact that the encapsulation process was effectively carried out, and other preencapsulation processes such as selection of excipients and granulation were also accurately done.

#### 3.2.4. *In Vitro* Dissolution Profile of Formulated Capsules

**Figure 1:**
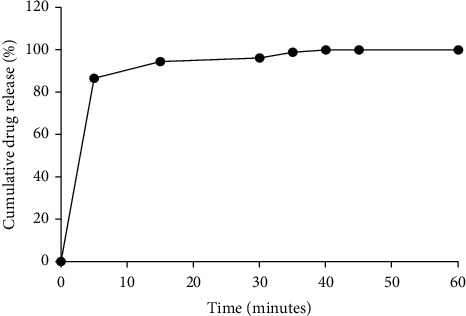
*In vitro* dissolution profile of formulated capsules.

Figure 1 shows the dissolution profile of the formulated capsules. According to [11], for nonmodified release dosage forms, not less than 70% of the active ingredient should be released by the 45^th^ minute of being in the dissolution medium. Based on the results obtained, by the 45^th^ minute, 98.46% of the active ingredient was released. This means that the formulated capsules passed the dissolution test and can be said to dissolve well in physiological solution to make the active ingredient or extract available for absorption and the desired pharmacological activity obtained [11, 13].

### 3.3. Evaluation of Formulated Suspension

#### 3.3.1. Appearance Test

In order to check for the presence or absence of undesirable physical changes such as aggregation, caking, and crystal growth formation, the formulated suspension was critically studied over a period of 4 weeks. These undesirable physical changes affect the aesthetic appeal of the formulation and gives an indication of physical instability [10, 20]. The formulated suspension was devoid of any undesirable physical changes (Table 4). This indicates that the suspension is physically stable and maintains its aesthetic appeal, and ultimately, the excipients selected for the formulation were ideal.

#### 3.3.2. Sedimentation Volume and Rate of Formulated Suspension

**Figure 2:**
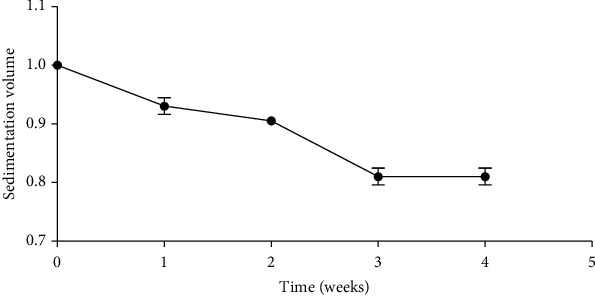
Sedimentation volume of formulated suspension.

A basic requirement of a good suspension is that the suspended particles should not settle quickly in order to enhance uniform and accurate dosing. An ideal suspension should have a high sedimentation volume usually within 0.5-1. The larger the value, the better the suspendability of the particles and the more stable the suspension [20–22]. The formulated suspension had a final and constant average sedimentation volume of 0.81 from the third (3) week through to the fourth (4) week (Figure 2). This high sedimentation volume confers good stability index on the formulated suspension and indicates that the suspension was well formulated. The high sedimentation volume also culminated in a very low sedimentation rate of 4.17 × 10^−6^ mL/sec for the formulated suspension. This low sedimentation rate means that the suspended particles do not settle easily, and hence, there will be ample time for the patient to take a consistent and an accurate dose of the formulation.

#### 3.3.3. pH, Rheology, and Redispersibility of Formulated Suspension

**Figure 3:**
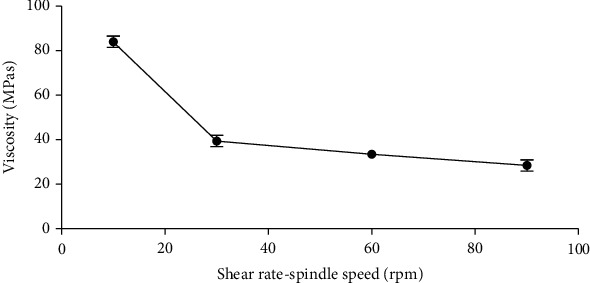
Effect of speed of rotation on the viscosity of formulated suspension.

The pH of the formulated suspension remained fairly constant with no significant difference (*p* ≥ 0.05) throughout the 4-week study period (Table 5). This indicates the absence of any physico-chemical change and confirms the stability of the formulated suspension [20, 23, 24]. A key attribute of a good pharmaceutical suspension is its easy pourability, and to achieve this, the flow time should be relatively short with a corresponding apparent viscosity [16, 20, 23]. This phenomenon was also observed in the formulated suspension (Table 5), an indication that the suspension can easily be poured from its primary package. Pharmaceutical suspensions are thermodynamically unstable, and hence, after settling of its solid particles, it is expected that the sediments generated should be easily and immediately redispersed upon shaking. A well formulated and stable suspension will require few strokes (<100) to redisperse its solid particles [25]. The formulated suspension required less than six (6) strokes to redisperse the solid particles over the period of study (Table 5). This further confirms that the suspension is a well-formulated one which possesses the ideal properties of a good suspension. The formulated suspension also exhibited a pseudoplastic flow (Figure 3) which implies that with minimal agitation, the suspension will be easily redispersed, and a stable dose can be withdrawn. This characteristic is necessary of an ideal suspension.

#### 3.3.4. Drug Content

According to [11], the amount of drug substance calculated should be within the range of 85.0% to 115.0% in nine of the dosage units assayed with no unit being out of the range of 75.0% to 125.0%. All sampled doses contained the required content of the active ingredient (Table 6), indicating that the suspension passed the drug content test. This implies that each volume will deliver the needed amount of the active ingredient and avoid overdosing and underdosing which are undesirable for the patient. This ultimately indicates that the processes involved in the formulation of the suspension were effectively and efficiently carried out.

#### 3.3.5. *In Vitro* Dissolution Profile of Formulated Suspension

**Figure 4:**
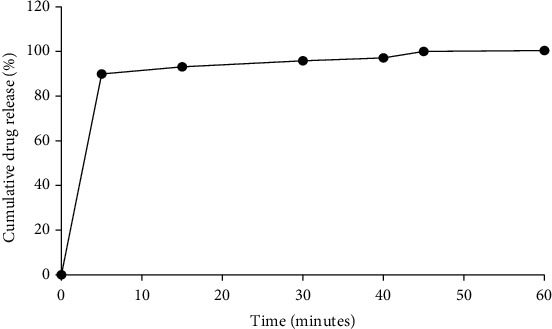
Dissolution profile of formulated suspension.

According to [11], for nonmodified release dosage forms, not less than 70% of the drug should be released by the 45th minute of being in the dissolution medium. Based on the release profile obtained in Figure 4, 99.95% of the extract had been released at the 45^th^ minute, an indication that the suspension passed the dissolution test. This implies that the suspension will be able to release the active ingredients within time for absorption of the active ingredients to occur and ultimately achieve the needed therapeutic effect [11, 19].

## 4. Conclusion

Suspension and capsules of the ethanolic extract of *Cola nitida* seeds have been successfully formulated. The formulated dosage forms met the pharmacopeia criteria for quality assessment and can be used as suitable alternatives in the management and treatment of diarrhea.

## Figures and Tables

**Table 1:** Weight uniformity of formulated capsules.

Number of capsules used	Average weight (g)	Number of capsules deviating by ±10%	Number of capsules deviating by ±20%
20	0.287	Nil	Nil

**Table 2:** Disintegration time of formulated capsules.

Disintegration test	Test 1	Test 2	Average test
Time (minutes)	2.05	2.12	2.085 ± 0.05

**Table 3:** Drug content of formulated capsules.

Capsule number	Absorbance	Drug content (%)
1	0.354	99.23
2	0.358	100.00
3	0.359	100.77
4	0.351	98.45
5	0.358	100.00
6	0.359	100.77
7	0.350	97.98
8	0.354	99.23
9	0.359	100.77
10	0.354	99.23

**Table 4:** Physical stability of formulated suspension.

Week	Physical instability (aggregation, caking, and crystal growth formation)
1	No physical instability observed
2	No physical instability observed
3	No physical instability observed
4	No physical instability observed

**Table 5:** pH, flow time, apparent viscosity, and redispersibility of formulated suspension.

Week	pH	Flow time (s)	Apparent viscosity (*η*) (mL/s)	Redispersibility (number of strokes)
0	6.76 ± 0.015^ns^	7.67 ± 0.58	1.305 ± 0.34	4
1	6.76 ± 0.010^ns^	8.00 ± 0.03	1.250 ± 0.07	5
2	6.75 ± 0.012^ns^	7.53 ± 0.50	1.331 ± 0.38	4
3	6.74 ± 0.010^ns^	7.64 ± 0.45	1.311 ± 0.45	4
4	6.74 ± 0.006^ns^	7.74 ± 0.25	1.296 ± 0.32	4

**Table 6:** Drug content of formulated suspension.

Sampling number for each 5 mL	Absorbance	Drug content (%)
1	0.325	90.47
2	0.345	96.15
3	0.347	96.92
4	0.354	99.14
5	0.340	95.12
6	0.347	96.92
7	0.345	96.15
8	0.352	98.19
9	0.353	98.46
10	0.359	100.50

## Data Availability

The data used to support the findings of this study are included in the article and also available from the corresponding author upon request.
